# Efficacy and Safety of Fuzi Formulae on the Treatment of Heart Failure as Complementary Therapy: A Systematic Review and Meta-Analysis of High-Quality Randomized Controlled Trials

**DOI:** 10.1155/2019/9728957

**Published:** 2019-12-23

**Authors:** Meng-Qi Yang, Yong-Mei Song, Huan-Yu Gao, Yi-Tao Xue

**Affiliations:** ^1^College for Traditional Chinese Medicine, Shandong University of Traditional Chinese Medicine, Jinan 250355, China; ^2^Institute for Literature and Culture of Chinese Medicine, Shandong University of Traditional Chinese Medicine, Jinan 250355, China; ^3^Department of Cardiology, Affiliated Hospital of Shandong University of Traditional Chinese Medicine, Jinan 250014, China

## Abstract

**Objective:**

Heart failure is a major public health problem worldwide nowadays. However, the morbidity, mortality, and awareness of heart failure are not satisfied as well as the status of current treatments. According to the standard treatment for chronic heart failure (CHFST), Fuzi (the seminal root of *Aconitum carmichaelii* Debx.) formulae are widely used as a complementary treatment for heart failure in clinical practice for a long time. We are aiming to assess the efficacy and safety of Fuzi formulae (FZF) on the treatment of heart failure according to high-quality randomized controlled trials (RCTs).

**Methods:**

RCTs in PubMed, Cochrane Library, China National Knowledge Infrastructure (CNKI), Chinese Scientific Journals Database (VIP), and Wanfang Database were searched from their inception until June 2019. In addition, the U.S. National Library of Medicine (clinicaltrials.gov) and the Chinese Clinical Trial Registry (http://www.chictr.org.cn) were also searched. We included RCTs that test the efficacy and safety of FZF for the treatment of heart failure, compared with placebo, CHFST, or placebo plus CHFST. The methodological quality of included studies were evaluated by the Cochrane Collaboration's tool for assessing risk of bias. RCTs with Cochrane risk of bias (RoB) score ≥4 were included in the analysis. The meta-analysis was conducted through RevMan 5.2 software. The GRADE approach was used to assess the quality of the evidence.

**Results:**

Twelve RCTs with 1490 participants were identified. The studies investigated the efficacy and safety of FZF, such as FZF plus the CHFST vs placebo plus CHFST (*n* = 4), FZF plus CHFST vs CHFST (*n* = 6), FZF plus digoxin tablets (DT) plus CHFST vs placebo plus DT plus CHFST (*n* = 1), and FZF plus placebo plus CHFST vs placebo plus DT plus CHFST (*n* = 1). Meta-analysis indicated that FZF have additional benefits based on the CHFST in reducing plasma NT-proBNP level, MLHFQ scores, Lee's heart failure scores (LHFs), and composite cardiac events (CCEs). Meanwhile, it also improved the efficacy on TCM symptoms (TCMs), NYHA functional classification (NYHAfc), 6MWD, and LVEF. Adverse events were reported in 6 out of 12 studies without significant statistical difference. However, after assessing the strength of evidence, it was found that only the quality of evidence for CCEs was high, and the others were either moderate or low or very low. So we could not draw confirmative conclusions on its additional benefits except CCEs. Further clinical trials should be well designed to avoid the issues that were identified in this study.

**Conclusion:**

The efficacy and additional benefits of FZF for CCEs were certain according to the high-quality evidence assessed through GRADE. However, the efficacy and additional benefits for the other outcomes were uncertain judging from current studies. In addition, the safety assessment has a great room for improvement. Thus, further research studies are needed to find more convincing proofs.

## 1. Background

Heart failure is a public health problem in clinical cardiology nowadays. There are about 3% to 5% people suffering from heart failure (HF) according to epidemiological surveys worldwide. The morbidity of HF is about 2% in developed countries and 1.3% in China, which means nearly 18 million people are having HF in China. Meanwhile, the proportion of people over 65 years with HF is about 10%, indicating that people are more likely to suffer from heart failure when they get older. The 1-year mortality rate is ranged from 20% to 40% in different countries in patients who are readmitted for heart failure, about 50% of HF patients died within five years after diagnosis, and the 10-year mortality is more than 90%. It is amazing that the mortality of HF is even higher than breast cancer, prostate cancer, colorectal cancer, and other common cancers. The mortality of lung cancer is only 18.4%, which is the highest in cancers. However, most of the patients with HF are still having a superficial knowledge on HF. Although HF is more common in the elderly, about 30% of the patients mistake the symptoms of HF as normal aging phenomena [1–5].

In general, current treatments for HF are relatively fixed, including diuretics, angiotensin-converting enzyme inhibitors or angiotensin receptor blockers, beta-blockers, aldosterone receptor antagonists, digitalis, and vasodilating agents, according to the guidelines for CHFST. However, these drugs only achieve good short-term effects. This is why the number of deaths and readmission resulting from HF continues to rise despite of the advances in drug treatment strategies for HF. From the perspective of TCM, the primary cause of HF is the yang deficiency of heart that results from Qi inadequacy and blood stasis in general consideration. Based on the primary cause of HF, many Chinese herbs have demonstrated safety and efficacy in the management of HF in both animal models and humans [6–12]. Fuzi is widely used in the treatment of HF as an adjuvant therapy in our long-term clinical practice whether in decoction or other dosage forms. Since Fuzi is a major Chinese herb for restoring yang for resuscitation, it is contained in FZF, which are made into various kinds of forms such as granule, capsule, pill, oral decoction, and injection for convenient use. It is beneficial to HF patients in relieving symptoms and improving indicators despite of its toxicity as known. This study aimed at investigating the efficacy and safety of FZF on the treatment of HF and providing reference for clinical diagnosis and treatment.

## 2. Methods

This systematic review and meta-analysis are based on the Preferred Reporting Items for Systematic Reviews and Meta-Analyses (PRISMA) statement search strategy [13].

### 2.1. Search Strategy

PubMed, Cochrane Library, China National Knowledge Infrastructure (CNKI), Chinese Scientific Journals Database (VIP), and Wanfang Database were retrieved. The following search terms were used and varied depending on which database was searched: “Heart failure,” “Cardiac Failure,” “Heart Decompensation,” “Decompensation, Heart,” “Heart Failure, Right-Sided,” “Heart Failure, Right Sided,” “Right-Sided Heart Failure,” “Right Sided Heart Failure,” “Myocardial Failure,” “Congestive Heart Failure,” “Heart Failure, Congestive,” “Heart Failure, Left-Sided,” “Heart Failure, Left Sided,” “Left-Sided Heart Failure,” “Left Sided Heart Failure,” “Traditional Chinese medicine,” “Chung I Hsueh,” “Hsueh, Chung I,” “Traditional Medicine, Chinese,” “Zhong Yi Xue,” “Chinese Traditional Medicine,” “Chinese Medicine, Traditional,” “Traditional Tongue Diagnosis,” “Tongue Diagnoses, Traditional,” “Tongue Diagnosis, Traditional,” “Traditional Tongue Diagnoses,” “Traditional Tongue Assessment,” “Tongue Assessment, Traditional,” and “Traditional Tongue Assessments”. The database was searched from their start date until June 2019. Conference proceedings and dissertations were also searched from CNKI, VIP, and Wanfang databases for unpublished trials. Moreover, we also manually searched additional relevant studies through the U.S. National Library of Medicine (clinicaltrials.gov) and The Chinese Clinical Trial Registry (http://www.chictr.org.cn). Specific herb name “Fuzi” was not specifically searched to ensure that eligible herbal formulae were included as much as possible.

### 2.2. Inclusion Criteria

1. Type of participants: researches involving adult patients with any type of HF.
2. Type of study: only RCTs that assessed the efficacy and safety of Fuzi for the treatment of HF were eligible.
3. Type of intervention: Fuzi must be included in the herbal formula used in the experimental group. There were no restrictions on the dosage forms of the drug (e.g., decoction, injection, pill, and capsule), dosage, frequency, or treatment time. Medications of the control group medications including placebo, CHFST, and placebo plus CHFST were also accepted.
4. Type of results: the efficacy of Fuzi on the treatment of HF was evaluated through primary outcomes of plasma NT-proBNP level and the efficacy of TCM symptoms (TCMs). Secondary outcomes included the efficacy of NYHA functional classification (NYHAfc), LVEF, 6-minute walk distance (6MWD), composite cardiac events (CCEs) such as death and readmission, the Minnesota Living with Heart Failure Questionnaire (MLHFQ) scores, and Lee's heart failure scores (LHFs). The safety was evaluated through adverse events and laboratory indexes.
5. RoB scored≥4 points [14].

### 2.3. Exclusion Criteria

If the above conditions were not met, the study was excluded. In addition, the following literatures were also excluded:

1. Duplicate publications
2. Animal experiments, mechanism, studies, reviews, protocols, experience, and case reports
3. Literatures on other TCM therapies, such as acupuncture, massage, moxibustion, Qi Gong, and Tai Chi

### 2.4. Study Selection

The titles and abstracts of the articles searched from the databases were read independently by two researchers in order to select the eligible RCTs. Full text of the studies that potentially met the predefined inclusion criteria were obtained and read. If there were some overlap or duplicate in the articles, only the most recent information was included. The disputes about the literature selection were resolved by discussing with the corresponding authors of this study.

### 2.5. Data Extraction

Two researchers extracted data from the eligible trials independently by use of predesigned standard data extraction forms. The following details were extracted: (1) publication year, the first authors' name, publication language, study design, investigational site, and the type of HF; (2) the characteristics of participants, including the number, sex, and mean age; (3) treatment information, including details of interventions management and course of treatment; and (4) outcome measurement and adverse effect. In studies with multiple comparison groups, the most relevant comparison group was chosen for the final analysis. If outcomes were at different time points of the study, the data of the last time point were extracted.

### 2.6. Quality Assessment

The methodological quality of the included studies was assessed by using the risk of bias (RoB) tools, provided by the Cochran's Systematic Review Handbook on interventions which include the following seven aspects: (A) random sequence generation (selection bias); (B) allocation concealment (selection bias); (C) blinding of participants and personnel (performance bias); (D) blinding of outcome assessment (detection bias); (E) incomplete outcome data (attrition bias); (F) selective reporting (reporting bias); and (G) other bias. Modified Jadad scale was also used to score included studies [15–17].

### 2.7. Fuzi Formulae Composition

The elements of FZF in each included study were recorded, such as the name, ingredients, and dosage of formula. The frequency used for specific herb was also calculated.

### 2.8. Data Analysis

Information from included studies was aggregated to produce a quantitative summary using the software Cochrane Collaboration Review Manage (RevMan 5.2). The Stata 12.0. Continuous data, such as plasma NT-proBNP level, LVEF, 6MWD, MLHFQ scores, and LHFs, were expressed as mean difference (MD) or standardized mean difference (SMD), whereas dichotomous data such as the efficacy of TCMs, NYHAfc, and CCEs were reported as relative risk (RR) with 95% confidence intervals (CIs). The statistical heterogeneity among trials was assessed using the chi-squared test and *I*^2^ statistic. If no heterogeneity exists (*P* > 0.05, *I*^2^ < 50%), a fixed effect model (FEM) was applied; otherwise, the random effect model (REM) was a more plausible match. However, since there were different ingredients in FZF, discrepancies in effect sizes should not be ignored. Therefore, whatever the heterogeneity index *I*^2^ was, we conducted REM to balance the effects of each study. Sensitivity analysis was performed by changing analysis combination to explore the impact of confounding factors. Meanwhile, in consideration of the differences in interventions and treatments, the subgroup analysis was performed using the *Z*-test. The differences between treatment groups and control groups were considered to be statistically significant when *P* < 0.05. If an outcome was reported in more than ten studies, funnel plots and Egger's test were used to examine their publication bias. Finally, we used the GRADE approach to access the strength of the evidence so as to make our results more credible.

## 3. Results

### 3.1. Description of Studies

**Figure 1:**
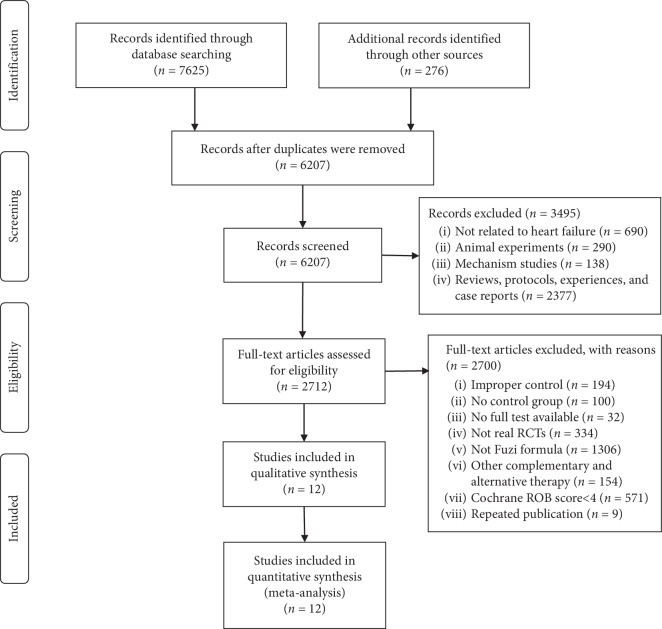
Prisma 2009 flow diagram.

A total of 7901 studies were retrieved from the five electronic databases and other sources. After removing the duplicate, 6207 records remained. By screening the titles and abstracts, 3495 records were excluded, among which 690 studies were not related to HF, 290 papers were animal experiments, 138 of them were mechanism studies, and 2377 papers were reviews, protocols, experience, or case reports. By reading the full text, 2700 studies were removed, including 194 studies with improper control interventions, 100 studies without control group, 32 studies without full text, 334 of them were unqualified, 1306 studies not using FZF, 154 studies containing other TCM therapies, such as acupuncture, massage, or scraping, and 571 studies with low methodological quality, and 9 studies as duplicates. Ultimately, 12 eligible studies with Cochrane RoB score ≥4 were included in this study [18–29]. A PRISMA flow diagram depicted the search process and study selection (Figure 1).

### 3.2. Study Characteristics

The characteristics of the 12 included trials are summarized in Table 1. All eligible studies were conducted in China. 2 articles were published in English [24, 29], and the rest were in Chinese [18–23, 25–28]. 3 were multicenter studies [19, 24, 29], and the others were single-center study [18, 20–23, 25–28]. Among the included studies, 9 were related to chronic heart failure (CHF) [18, 19, 21–26, 29], 2 were related to diastolic heart failure (DHF) [20, 27], and 1 was related to systolic heart failure [27]. The sample size of the included studies ranged from 60 to 491, enrolling a total of 1490 participants, 735 patients in experimental groups, and 755 patients in control groups. All of the 12 RCTs were two arms. 6 studies compared FZF plus CHFST with CHFST [18, 21, 25–28], 4 studies compared FZF plus CHFST with placebo plus CHFST [20, 22–24, 29], 1 study compared FZF plus digoxin tablets (DT) plus CHFST with placebo plus DT plus CHFST [22], and the last one compared FZF plus placebo plus CHFST with placebo plus DT plus CHFST [19]. The preparations used in the 12 RCTs were administered orally in decoction (4 comparisons) [22, 26–28], granules (2 comparisons) [19, 21], capsules (4 comparisons) [18, 20, 23, 24], pills (1 comparison) [25], and injections (1 comparison) [29]. The treatment duration ranged from 7 ± 1 days to 9 months.

### 3.3. Description of Fuzi Formulae

The constituents of FZF in included studies are detailed in Table 2. Thirty herbs were used in the twelve different FZF. The top 6 frequently used herbs were *Aconitum carmichaelii* Debx. (Aconiti lateralis radix preparata), *Panax ginseng* C.A. Mey (Ginseng radix et rhizome), *Salvia miltiorrhiza* Bge. (Salvia miltiorrhiza), *Poria cocos* (Schw.), *Wolf* (Tuckahoe), *Astragalus membranaceus* (Fisch.) Bge.var.mongholicus (Bge.), *Hsiao astragalus membranaceus* (Fisch.) Bge. (fresh Mongolian milkvetch root), and *Descurainia Sophia* (L.) Webb.ex Prantl. (Semen Descurainia lepidii), which were used at least 4 times (Table 3).

### 3.4. RoB Assessment

The RoB evaluation is shown in Table 4. All the included studies were described as “randomized” with appropriate methods of sequence generation, such as random number table (8 studies) [18, 20, 21, 24–28], Statistical Analysis System (SAS) software (1 study) [22], central assignment (1 study) [29], Package for Encyclopaedia of Medical Statistics3.1 (PEMS3.1) software (1 study) [23], and computer-generated stochastic system (1 study) [19]. The RoB of the 12 studies were low in the domain of sequence generation. 1 study applied “sealed envelopes” [23], and 6 studies mentioned double blindness [19, 20, 23, 24, 26, 29]. 10 studies had described dropouts and provided adequate explanations or appropriate methods to treat missing data [18–20, 22–25, 27–29]. Two studies did not mention dropouts [21, 26]. No significant other bias was found in the included studies. Finally, among the 12 studies, 7 articles were scored 4 points [18, 20, 21, 25–28], 1 article was scored 5 points [29], and the other 4 studies were scored 6 points according to the revised Jadad scale [19, 22–24].

### 3.5. Primary Outcomes

#### 3.5.1. Plasma NT-proBNP Level

**Figure 2:**
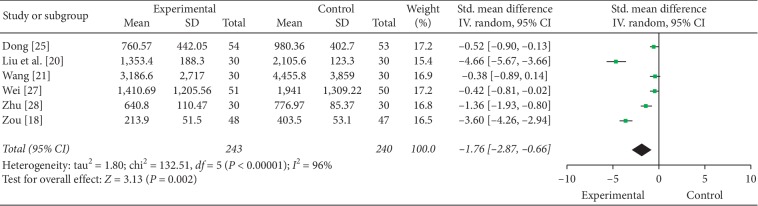
The forest plot of plasma NT-proBNP level.

6 studies evaluated the plasma NT-proBNP level, and a reduction was showed in FZF plus CHFST, compared with CHFST (SMD = −1.76, 95% CI: −2.87 to −0.66, *P*=0.002, heterogeneity *χ*2 = 132.51, *P* < 0.00001, *I*^2^ = 96%, Figure 2) [18, 21, 25–28]. However, the quality of the evidence was low (Table 5). So further research is very likely to have an important impact on our confidence in the estimate of effect and is likely to change the estimate. The result of the heterogeneity test was 96%, which indicated they had high heterogeneity, so the statistical analysis was conducted with REM.

#### 3.5.2. Efficacy of TCM Symptoms

**Figure 3:**
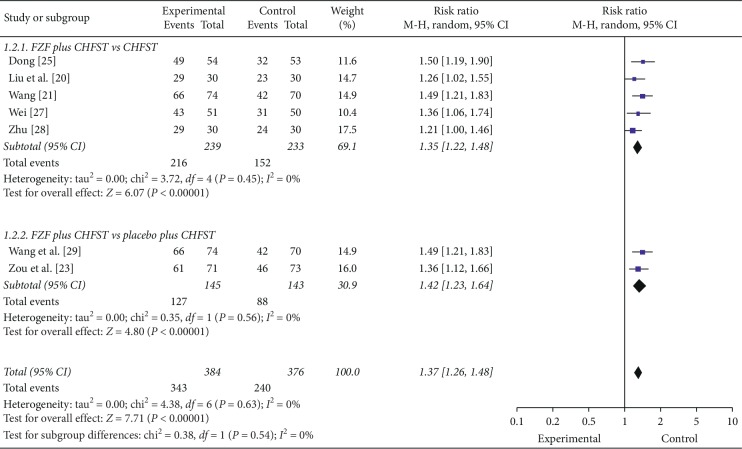
The forest plot of efficacy of TCMs.

9 trials reported the efficacy of TCM symptoms. Meta-analysis showed that FZF were better at improving the efficacy of TCM symptoms both in subgroups that compared FZF plus CHFST with CHFST (RR = 1.35, 95% CI: 1.22 to 1.48, *P* < 0.00001, heterogeneity *χ*2 = 3.72, *P*=0.45, *I*^2^ = 0%, Figure 3) [21, 25–28] and FZF plus CHFST with placebo plus CHFST (RR = 1.42, 95% CI: 1.23 to 1.64, *P* < 0.00001, heterogeneity *χ*2 = 0.35, *P*=0.56, *I*^2^ = 0%, Figure 3) [23, 29]. The comparison of FZF plus DT plus CHFST with placebo plus DT plus CHFST [22] and FZF plus placebo plus CHFST compared with placebo plus DT plus CHFST [19] demonstrated that FZF combined with CHFST treatment had equivalent efficacy compared to DT combined with CHFST treatment, as well as FZF combined with low-dose DT (*P* > 0.05). The quality of the evidence of the above subgroups were moderate and high, respectively (Table 5). Further research studies are likely to have an important impact on our confidence in the estimate of effect and may change the estimate of the subgroup of FZF plus CHFST with CHFST, while the subgroup of FZF plus CHFST with placebo plus CHFST are much better. Above all, the efficacy of TCMs was uncertain according to current evidence.

### 3.6. Secondary Outcomes

#### 3.6.1. NYHA Functional Classification (NYHAfc)

**Figure 4:**
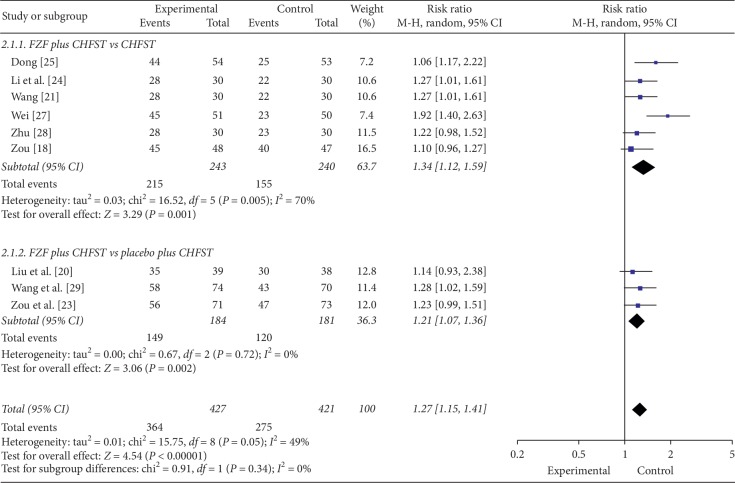
The forest plot of NYHA functional classification (NYHAfc).

10 trials reported the efficacy on NYHAfc as outcome. Meta-analysis showed that FZF were better at reducing the NYHAfc in both subgroups. The subgroups were FZF plus CHFST with CHFST (RR = 1.34, 95% CI: 1.12 to 1.59, *P*=0.001, heterogeneity *χ*2 = 16.52, *P*=0.005, *I*^2^ = 70%, Figure 4) [18, 21, 24–28] and FZF plus CHFST with placebo plus CHFST (RR = 1.21, 95% CI: 1.07 to 1.36, *P*=0.002, heterogeneity *χ*2 = 0.67, *P*=0.72, *I*^2^ = 0%, Figure 4) [23, 29]. There was no significant homogeneity of this outcome in the overall effect (RR = 1.27, 95% CI: 1.15 to 1.41, *P* < 0.00001, heterogeneity *χ*2 = 5.75, *P*=0.05, *I*^2^ = 49%, Figure 4). The comparison of FZF plus DT plus CHFST with placebo plus DT plus CHFST demonstrated that FZF had had equivalent efficacy compared to DT at the base of combined treatment (*P* > 0.05). The quality of the evidence of the above subgroups was low and moderate, respectively (Table 6). So the efficacy on NYHAfc was uncertain according to current evidence.

#### 3.6.2. LVEF

**Figure 5:**
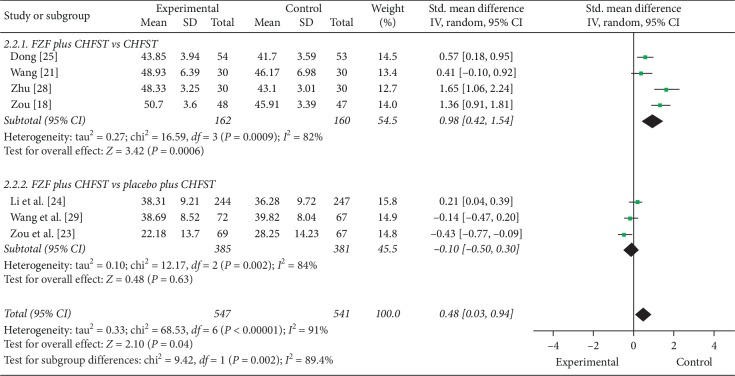
The forest plot of echocardiography measurements (LVEF).

7 trials reported LVEF as outcome. Meta-analysis demonstrated that FZF were better at improving LVEF. The two subgroups compared FZF plus CHFST with CHFST (SMD = 0.98, 95% CI: 0.42 to 1.54, *P*=0.0006, heterogeneity *χ*2 = 16.59, *P*=0.0009, *I*^2^ = 82%, Figure 5) [18, 21, 25, 28] and FZF plus CHFST with placebo plus CHFST (SMD = −0.10, 95% CI: −0.50 to 0.30, *P*=0.63, heterogeneity *χ*2 = 12.17, *P*=0.002, *I*^2^ = 84%, Figure 5) [23, 24, 29]. There was high homogeneity of this outcome in the overall effect (SMD = 0.48, 95% CI: 0.03 to 0.94, *P*=0.004, heterogeneity *χ*2 = 68.53, *P* < 0.00001, *I*^2^ = 91%, Figure 5). However, the quality of the evidence for this outcome was low. As for subgroups above, the strength of their evidence was very low and moderate, respectively (Table 6). We were very uncertain about the estimate of this outcome; however, the comparison of FZF plus CHFST with placebo plus CHFST deserved more further researches.

#### 3.6.3. 6MWD

**Figure 6:**
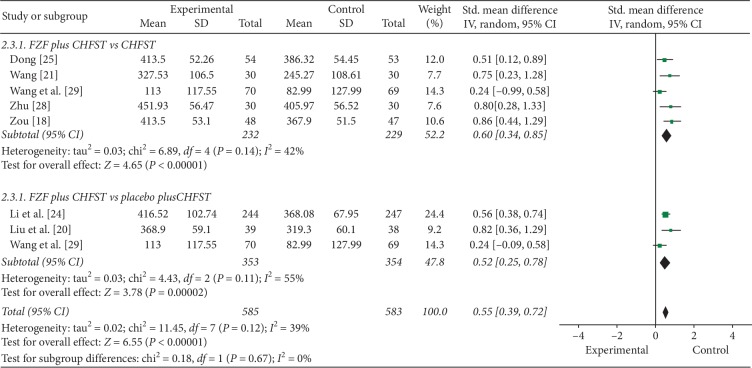
The forest plot of 6MWD.

7 trials with 8 comparisons reported 6MWD as outcome. Meta-analysis showed that FZF were better at improving the 6MWD in all subgroups. The subgroups compared FZF plus CHFST with CHFST (SMD = 0.60, 95% CI: 0.34 to 0.85, *P* < 0.00001, heterogeneity *χ*2 = 6.89, *P*=0.14, *I*^2^ = 42%, Figure 6) [18, 21, 25, 28, 29] and FZF plus CHFST with placebo plus CHFST (SMD = 0.52, 95% CI 0.25 to 0.78, *P*=0.0002, heterogeneity *χ*2 = 4.43, *P*=0.11, *I*^2^ = 55%, Figure 6) [20, 24, 29]. There was no homogeneity of this outcome in the overall effect (SMD = 0.55, 95% CI: 0.39 to 0.72, *P* < 0.00001, heterogeneity *χ*2 = 11.45, *P*=0.12, *I*^2^ = 39%, Figure 6). Although the results indicated a benefit in the FZF overall, the beneficial results were uncertain despite the moderate quality of the evidence (Table 6).

#### 3.6.4. MLHFQ Scores and Lee's Heart Failure Scores

**Figure 7:**
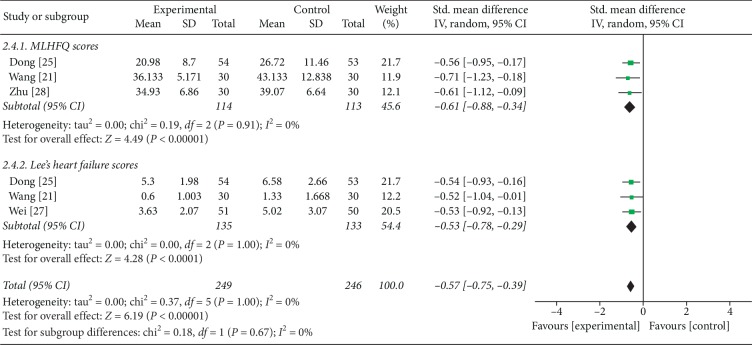
The forest plot of MLHFQ scores and Lee's heart failure scores.

4 trials with 6 comparisons reported MLHFQ scores (MLHFQs) and Lee's heart failure scores (LHFs) as outcome. Meta-analysis showed that FZF were better at reducing MLHFQs in the subgroup which comparing FZF plus CHFST with CHFST (SMD = −0.61, 95% CI: −0.88 to −0.34, *P* < 0.00001, heterogeneity *χ*2 = 0.19, *P*=0.91, *I*^2^ = 0%, Figure 7) [21, 25, 28]. The LHFs were also improved according to the comparison of FZF plus CHFST with CHFST (SMD = −0.53, 95% CI: −0.78 to −0.29, *P* < 0.0001, heterogeneity *χ*2 = 0.37, *P*=1.00, *I*^2^ = 0%, Figure 7) [21, 25, 27]. Despite the quality of the evidence for the subgroups being low (Table 6), the beneficial results were uncertain and might been changed by further well-designed researches.

#### 3.6.5. CCEs

**Figure 8:**
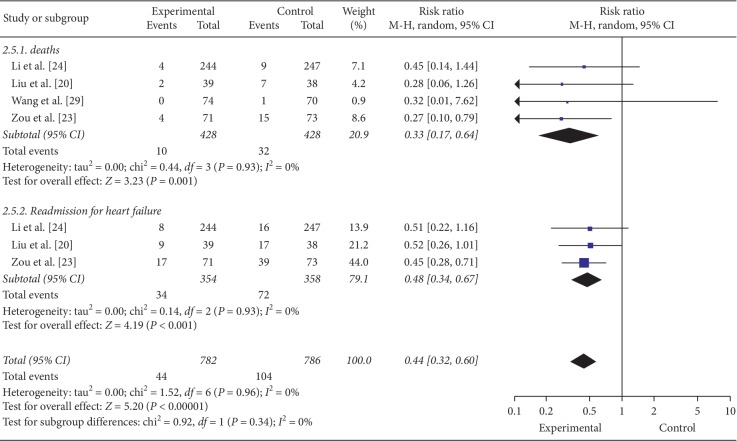
The forest plot of CCEs.

4 trials with 7 comparisons reported CCEs as outcome. Meta-analysis showed that FZF were better at reducing death after the comparison of FZF plus CHFST with placebo plus CHFST (RR = 0.33, 95% CI: 0.17 to 0.64, *P*=0.001, heterogeneity *χ*2 = 0.44, *P*=0.93, *I*^2^ = 0%, Figure 8) [20, 23, 24, 29]. Readmission was also reduced according to the comparison of FZF plus CHFST with placebo plus CHFST (RR = 0.48, 95% CI 0.34 to 0.67, *P* < 0.0001, heterogeneity *χ*2 = 0.14, *P*=0.93, *I*^2^ = 0%, Figure 8) [20, 23, 24]. Moreover, the quality of evidence was high according to the GRADE approach (Table 6).

### 3.7. Adverse event(s)

**Figure 9:**
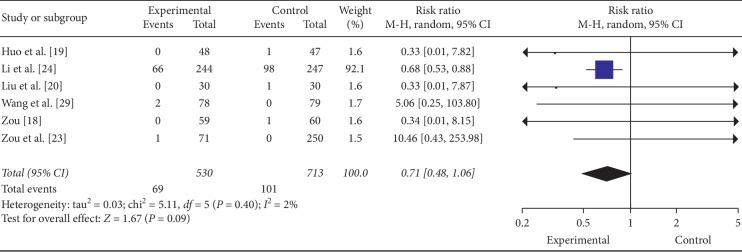
The forest plot of safety.

6 studies [18–20, 23, 24, 29] reported adverse events occurring during the treatment period, and a total of 9.4% (69/735) patients in the experimental groups and 13.4% (101/755) patients in control groups suffered from adverse events (Table 7). Some patients had more than one event. 6 studies [21, 22, 25–28] stated no adverse events happened during the treatment period. 4 studies [18–20, 23] provided adequate information of the adverse events. 3 studies reported erythra as adverse event in control groups [19, 20, 29], and the erythra was considered to be anaphylactic reaction to uncertain western medicine. 1 study reported cough as an adverse event in the control group [23], and the cough could be self-remission after medicine withdrawal. 1 study reported chest tightness and heart palpitation as adverse events in the control group [18], and the reason of these adverse events was not mentioned in detail. 1 study reported chills as an adverse event in the control group [29]. The last study did not mention adverse events, but there was no detailed description [24]. No significant abnormal was found in the blood routine, urine routine, liver function, and kidney function test. 1 study reported adverse events related to study drugs, and 20 cases in the experimental group and 23 cases in the control group were without a detail description [24]. This study also described arterial occlusive diseases (1 case in the control group), worsening heart failure (4 cases in the experimental group, and 7 cases in the control group), stroke (1 case in the experimental group and 1 case in the control group), lumbar fracture (1 case in the experimental group), and unknown reasons (2 cases in the experimental group and 3 cases in the control group) as serious adverse events. There was no report of any serious adverse events related to the study drugs. Three studies [21, 28, 29] reported safety with specific laboratory index, and there was no statistical significance. However, meta-analysis showed the safety of FZF was not satisfied, which needs to be improved in further studies (RR = 0.71, 95% CI: 0.48 to 1.06, *P*=0.09, heterogeneity *χ*2 = 5.11, *P*=0.40, *I*^2^ = 2%, Figure 9) [18, 20, 22–24, 29].

### 3.8. Publication Bias

**Figure 10:**
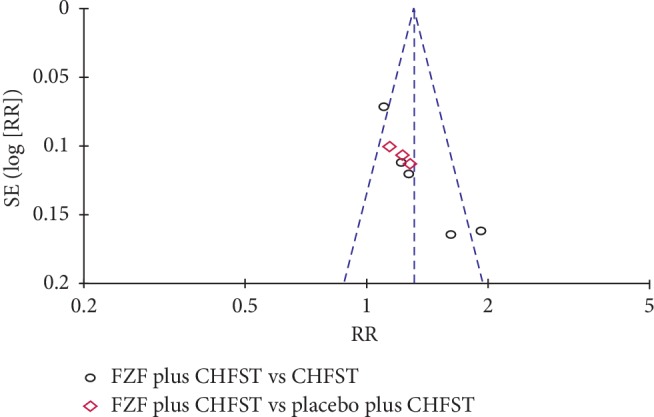
Funnel plot of NYHAfc.

**Figure 11:**
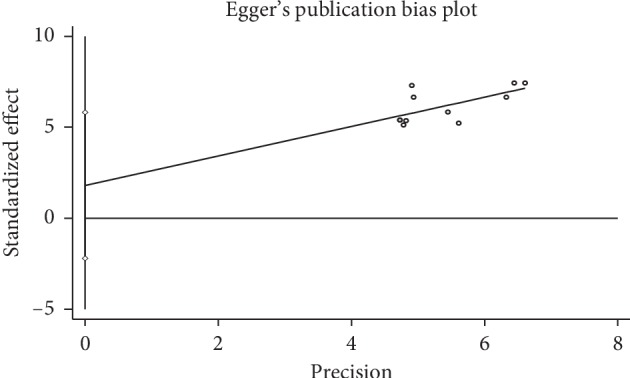
Egger's test of NYHAfc.

Funnel plots were reviewed on NYHAfc (Figure 10). The results showed symmetrical distribution of the outcomes of the efficacy of NYHAfc with Egger's test (*P*=0.335, *P* > 0.05) (Figure 11). Because the number of studies on the other outcomes are all less than 10, funnel plot and Egger's test were not appropriate.

### 3.9. Sensitivity Analysis

The sensitivity analysis did not indicate that the results of any individual study would change the final outcome, which meant that none of the studies can significantly affect the pooled OR and 95% CI.

## 4. Discussion

### 4.1. Summary of Evidence

The studies of TCM for the treatment of HF have been carried out for quite a long while and most of them, including animal experiments, clinical trials, or pharmacological studies, have indicated the efficacy of TCM at the basis of CHFST [30–32]. Fuzi are widely used as a TCM herb in the formulae of TCM based on the essence of heart-yang deficiency of heart failure, such as various decoctions made according to the principles of TCM syndrome, injections, and Chinese patent medicines [18, 19, 24, 25, 33–37]. However, the poor methodological quality and small sample sizes prevented the author from making firm conclusions. Despite the current evidence and wide application of FZF in clinical practice, our systematic review tried to determine the efficacy and safety of FZF on heart failure through analyzing 12 high-quality RCTs with 1490 participants. The present study indicates that FZF provide statistical benefits in improving the efficacy on NYHAfc and LVEF and reducing plasma NT-proBNP level as well. Furthermore, they can also improve the patients' prognosis and life quality and reduce the risks of patients in death and readmission for heart failure. However, despite the low strength of current evidence, the benefits were almost uncertain except the benefits for death and readmisssion. In addition, FZF appeared to be generally safe and well tolerated with mild adverse reactions. Although 6 reported adverse events, only 45 out of 1490 cases (3.0%) had adverse events possibly related to FZF without powerful evidence. No significant differences were found on laboratory indicators. Current evidence supported that FZF could be an adjuvant therapy for the treatment of heart failure on the basis of CHFST in improving death and readmission.

### 4.2. Limitations

There are several limitations in our primary studies as well. Firstly, the participants included were all with chronic heart failure. Therefore, the efficacy of FZF for acute heart failure are not clear. Thus, further research studies on FZF for the treatments of acute heart failure are needed. And the sample sizes of some studies were small, which might have influence on the results. Meanwhile, because all the participants in the studies came from China, the ethnic differences and regional differences were indefinite. Secondly, the components of FZF varied in producing area, species, processed methods, dosages, forms, and decocting. For the processed methods, 1 study used Heishunpian [24], 1 study used Baifupian [27], and 1 study used Fupian [22] in FZF, while the other studies used Fuzi [19, 25, 26, 28, 29], Shufuzi [18, 20, 23], and Paofuzi [21] as described. As for the decoction methods, 3 FZF needed to be decocted, 1 FZF was not described specifically [27], and only 1 study described the decoction method clearly [28]. Current studies indicate that decocting methods have some effects on the toxicity of Fuzi and different dosages may also have unknown influences uncover [38, 39]. Thirdly, although we included the high-quality RCTs according to a cumulative score of at least 4 out of 7 based on the Cochrane RoB tool domains and revised Jadad scale, the methodological details were still not adequate in some studies. Only 1 study [23] described a proper method of allocation concealment, and 6 studies [19, 20, 23, 24, 26, 29] employed the blinding procedure. Some studies were unable to be blinded, due to the fact that TCM is special in color, smell, and taste, which were difficult to be changed or covered. And most kinds of FZF were so different from western medicine, which cannot be ignored. Furthermore, current designs for RCTs could not meet the needs of blinding because of condition limitations. Six studies used placebo to replace FZF [19, 20, 22–24, 29], only one study described the specific composition of placebo, [20], and the capsules of placebo with similar appearance and the taste were made from lactose, dextrin, caramel, and edible pigments. One study replaced Shenfu injection (SFJ) with glucose injection. However, no study used a double-dummy technique to reduce the difference of drugs between the experiment and control groups. Improper blinding methods made it difficult to get certain results intentionally or unintentionally, and it is hard to ensure the credibility of study conclusions [40]. In addition, the intervention of trials with inadequate allocation concealment is 18% more “beneficial” than trials with adequate concealment [41]; however, lacking allocation concealment was common. Fourthly, due to traditional culture and the barrier of language, all RCTs were in English or in Chinese and were conducted in Chinese population, which restricts the generalizability of the findings. Finally, the strength of evidence was almost poor according to the GRADE approach, which made the final results less credible.

### 4.3. Implications for Practice

Modern pharmacological studies on Fuzi were performed (the seminal root of *Aconitum carmichaelii* Debx) to explain its mechanisms of actions. Fuzi was first recorded in “Shen Nong Ben Cao Jing,” which was known as one of the TCM classics for over thousands of years. The herb is good at restoring yang for resuscitation, tonifying fire, and helping yang, removing rheumatism and relieving pain because of its properties: pungent and sweet in flavor, pretty hot in nature, and extremely poisonous especially when the fresh herb was used [42]. So processed products of Fuzi with less toxic effect are commonly used nowadays. The common processed products including Yanfuzi, Heishunpian, Baifupian, Paofuzi, Danfuzi, Shufuzi, Weifuzi, and other decocting pieces processed by ginger juice, glycyrrhiza juice, tofu, etc. The main chemical components of Fuzi are aconitum alkaloids including C-19 diterpenoid alkaloids (aconitine, mesaconitine, and hypaconitin), C-20 diterpenoid alkaloids (songorine and songoramine), and nonalkaloids such as urical, *β*-sitosterol, daucosterol, and glyceryl monopalmitate. And the biological activities of Fuzi include enhancing myocardial systole, resisting inflammation, relieving pain, resisting tumor, promoting immunity, and influencing metabolism [43]. Most processed products of Fuzi could reduce its' poisonousness, and the chemical components of Fuzi are also changed indeed. Firstly, the toxicity of aconite is significantly reduced. Because aconitum alkaloids contain ester bonds and have thermal instability, and they are hydrolyzed to form monoester alkaloids and protoalkaloids with less toxicity after boiling. Meanwhile, aconitum alkaloids are prone to pinacol rearrangement and pyro-type alkaloids under acidic and heating conditions. The toxicity of aconitum alkaloids is relatively low, and their analgesic and anti-inflammatory effects are still obvious. In addition, the number of fatty alkaloids in Fuzi increased after running and the substitution of long chain fatty acyl and acetyl groups reduced the toxicity greatly [44, 45]. In a word, the toxicity of dicarboxylic alkaloids with high toxicity is reduced, and other alkaloids with low toxicity or nontoxicity are increased, so the toxicity of processed Fuzi is greatly reduced. Furthermore, the processed products of Fuzi will not only reduce its cardiotonic, analgesic, and anti-inflammatory effects but also increase the safe dose of Fuzi [46, 47]. Although many scholars have proved that Fuzi has a cardiotonic effect on different animal models of HF with its different preparations; further research studies are still needed to clarify the nature of the ingredients of the mixture and the mechanisms of action of different processed Fuzi products.

### 4.4. Implications for Further Studies

At first, we suggest that the protocol of clinical trials must be registered in clinical trials registry platform such as The U.S. National Library of Medicine (clinicaltrials.gov) and The Chinese Clinical Trial Registry (http://www.chictr.org.cn). Meanwhile, CONSORT 2010 statement should be applied in trial reporting and publication in order to draw normative conclusions for further studies. Secondly, in order to facilitate more reliable comparison of study results, the clinical trials must be well designed according to international standards. The enrollment of participants should be more wide, sample size needs to be appropriately calculated, the randomization principle and allocation concealment should be implemented with more attention, and the standards of measurement results need to be as uniform as possible, as well as the test medication, in order to strengthen the evidence and make results more reliable. The type of acute heart failure should be further studied, which could give precise evidence for clinic. Thirdly, Fuzi, Renshen, Danshen, Fuling, Huangqi, and Tinglizi were the most frequently used herbs in treating HF, which should be considered firstly when formulating optimal formula. Finally, there are large spaces on the exact pathomechanism of migraine, and the pharmacological mechanism of Fuzi remains largely unknown, which should be further investigated.

## 5. Conclusion

The efficacy and additional benefits of FZF for CCEs were certain according to the high-quality evidence assessed through GRADE. However, the efficacy and additional benefits for the other outcomes were uncertain based on current studies. Furthermore, the safety assessment has a great room for improvement. Thus, further researches are needed to find more convincing proof.

## Figures and Tables

**Table 1:** Basic characteristics of the included studies.

Included trial	Publication language	Study design and investigational sites	Type of HF	No. of participants (male/female; age years)		Intervention		Outcome index	Intergroup difference
				Experimental	Control	Experimental	Control		
Huo et al. [19]	Chinese	RCT, multicenter, China	CHF	26/34; 18–75 64.55 ± 7.33	31/28, 18–75 61.95 ± 8.92	CHFST plus Huaxinsu granules (11 g, tid) plus placebo (0.125 mg qd) for 4 weeks	CHFST plus placebo (11 g, tid) plus DT (0.125 mg qd) for 4 weeks	(1) Efficacy on western medicinal symptoms (2) Efficacy of TCM symptoms (3) Adverse event	(1) *P* > 0.05 (2) *P* > 0.05 (3) *P* > 0.05

Zou [18]	Chinese	RCT, single-center, China	CHF	21/29; 63 ± 8.2	23/27; 64 ± 6.6	CHFST plus Nuanxin capsules (1.35 g tid) for 6 months	CHFST for 6 months	(1) NYHAfc (2) Efficacy on TCM symptoms (3) Readmission (4) LVEF and LVED (5) 6MWD (6) NT-proBNP (7) Adverse events	(1) *P* < 0.05 (2) *P* < 0.05 (3) *P* < 0.05 (4) *P* < 0.05 (5) *P* < 0.05 (6) *P* < 0.05 (7) —
Liu et al. [20]	Chinese	RCT, single-center, China	DHF	23/18; 67.7 ± 8.1	39/24; 66.1 ± 8.8	CHFST plus Nuanxin capsules (1.35 g tid) for 9 months	CHFST plus placebo (1.35 g tid) for 9 months	(1) Efficacy on NYHAfc (2) TCM symptom scores (3) Readmission and death (4) 6MWD (5) Adverse events	(1) *P* < 0.05 (2) *P* < 0.05 (3) *P* < 0.05 (4) *P* < 0.05 (5) —

Wang [21]	Chinese	RCT, single-center, China	CHF	14/16; 52–75 72.33 ± 6.013	20/10; 40–75 61.67 ± 10.949	CHFST plus Tongyang Huoxue decoction (9 g bid) for 3 weeks	CHFST for 3 weeks	(1) Efficacy on TCM symptoms (2) TCM symptom scores (3) Efficacy of NYHAfc (4) Efficacy on LHFs (5) TCM symptom scores (6) Efficacy on 6MWT and 6MWD (7) LVEF (8) NT-proBNP (9) MLHFQs (10) MOS SF-36 scores (11) BP and HR (12) Safety and adverse events	(1) *P* < 0.05 (2) *P* < 0.05 (3) *P* > 0.05 (4) *P* < 0.05 (5) *P* < 0.05 (6) *P* < 0.05 (7) *P* < 0.05 (8) *P* < 0.05 (9) *P* < 0.05 (10) *P* < 0.05 (11) *P* > 0.05 (12) *P* > 0.05

Cao et al. [22]	Chinese	RCT, single-center, China	CHF	Total: 39/61; 36–77/25–82 66.04 ± 9.01/65.10 ± 8.74		CHFST plus Kangshuai decoction (10 ml tid) plus DT (0.125 mg qd) for 14 days	CHFST plus placebo (10 ml tid) plus DT (0.125 mg qd) for 14 days	(1) Efficacy on LHFs (2) Efficacy on NYHAfc (3) Efficacy on TCM symptoms (4) LVED/LVEF/SV	(1) *P* > 0.05 (2) *P* > 0.05 (3) *P* > 0.05 (4) *P* > 0.05

Zou et al. [23]	Chinese	RCT, single-center, China	CHF	32/39; 69.35 ± 1(2) 26	37/36; 70.06 ± 10.32	CHFST plus Nuanxin capsules (1.35 g tid) for 24 weeks	CHFST plus placebo capsule (1.35 g tid) for 24 weeks	(1) Efficacy on TCM symptoms (2) Efficacy on NYHAfc (3) MLHFQs (4) LVEF (5) Readmission (6) Death (7) Safety	(1) *P* < 0.05 (2) *P* < 0.05 (3) *P* < 0.05 (4) *P* < 0.05 (4) *P* < 0.05 (6) *P* > 0.05 (7) —

Li et al. [24]	English	RCT, multicenter, China	CHF	182/62 56.98 ± 11.59	188/59; 57.53 ± 11.05	CHFST plus qili qiangxin capsule (4 granules tid) for 12 weeks	CHFST plus placebo capsules (4 granules tid) for 12 weeks	(1) NT-proBNP (2) CCEs (3) NYHAfc (4) LVEF/LVED (5) 6MWD (6) MLHFQs (7) Adverse event(s)	(1) *P* < 0.05 (2) *P* < 0.05 (3) *P* > 0.05 (4) *P* < 0.05 (5) *P* < 0.05 (6) *P* < 0.05 (7) *P* > 0.05

Dong [25]	Chinese	RCT, single-center, China	CHF	30/24; 45–74 60.38 ± 5.41	29/24; 49–75 59.92 ± 5.41	CHFST plus Shenfu cardiac pill (2 pills tid) for 3 months	CHFST for 3 months	(1) TCM symptom scores (2) Efficacy on TCM symptoms (3) Lee's heart failure scores (4) Efficacy on heart failure scores (5) NYHAfc (6) MLHFQs (7) LVEF and LVED (8) NT-proBNP (9) 6MWD (10) Safety	(1). *P* < 0.05 (2) *P* < 0.05 (3) *P* < 0.05 (4) *P* < 0.05 (5) *P* < 0.05 (6) *P* < 0.05 (7) *P* < 0.05 (8) *P* < 0.05 (9) *P* < 0.05 (10) —

Li et al. [26]	Chinese	RCT, single-center, China	CHF	13/17; 30–85 61.45 ± 4.55	15/15; 32–83 60.65 ± 4.35	CHFST plus Baoyuan Shipi decoction (one dose qd) for 1 weeks	CHFST for 1 weeks	(1) Efficacy on TCM symptoms (2) Efficacy on NYHAfc (3) NT-proBNP (4) Safety	(1) *P* < 0.05 (2) *P* < 0.05 (3) *P* < 0.05 (4) —

Wei [27]	Chinese	RCT, single-center, China	DHF/SHF	20/31; 74.33 ± 5.40	24/26; 75.84 ± 4.02	CHFST plus Shenfu Jiuxin decoction (130 ml tid) for 10 days	CHFST for 10 days	(1) TCM symptom scores (2) Efficacy on TCM symptoms (3) Efficacy on NYHAfc (4) Lee's heart failure scores (5) Efficacy on LHFs (6) NT-proBNP	(1) *P* < 0.05 (2) *P* < 0.05 (3) *P* < 0.05 (4) *P* < 0.05 (5) *P* < 0.05 (6) *P* < 0.05

Zhu [28]	Chinese	RCT, single-center, China	SHF	14/16; 56.43 ± 9.85	13/17; 56.37 ± 10.01	CHFST plus Yiqi qiangxin decoction (150 ml bid) for 2 weeks	CHFST for 2 weeks	(1) Efficacy on NYHAfc (2) TCM symptom scores (3) Efficacy on TCM symptoms (4) MLHFQs (5) LVEF (6) NT-proBNP (7) 6MWT (8) Safety	(1) *P* < 0.05 (2) *P* < 0.05 (3) *P* < 0.05 (4) *P* < 0.05 (5) *P* < 0.05 (6) *P* < 0.05 (7) *P* < 0.05 (8) —

Wang et al. [29]	English	RCT, multicenter, China	CHF	42/32; 68.58 ± 8.42	48/22; 68.14 ± 8.73	CHFST plus SFJ for 7 ± 1 days	CHFST plus placebo for 7 ± 1 days	(1) Efficacy of NYHAfc (2) Efficacy of TCM syndrome scores (3) Efficiency of LHFs (4) 6MED (5) LVEF (6) Death (7) Safety (8) Laboratory indexes	(1) *P* < 0.05 (2) *P* < 0.05 (3) *P* < 0.05 (4) *P* < 0.05 (5) *P* > 0.05 (6) *P* > 0.05 (7) *P* > 0.05 (8) *P* > 0.05

RCT = randomized controlled trial; CHF = chronic heart failure; SHF = systolic heart failure; DSH = diastolic heart failure; CHFST = standard treatment of heart failure; DT = digoxin tablets; SFD = Shenfu decoction; SFJ = Shenfu injection; NYHAfc = NYHA functional classification; LHFs = Lee's heart failure scores; MLHFQs = Minnesota Living with Heart Failure Questionnaire scores; CCEs = composite cardiac events.

**Table 2:** The element of Fuzi formula in each included study.

Included trials	Formula	Ingredient			Dosage (g)
		Latin name	English name	Chinese name	
Huo et al. [19]	Huaxinsu granule	(1) *Aconitum carmichaelii* Debx. (2) *Astragalus membranaceus* (Fisch.) Bge.var.mongholicus (Bge.) Hsiao and *Astragalus membranaceus* (Fisch.) Bge. (3) *Salvia miltiorrhiza* Bge. (4) *Paeonia lactiflora* Pall. (5) *Ilex pubescens* Hook et Arn. (6) *Plantago asiatica* L. (7) *Descurainia sophia* (L.) Webb ex Prantl. (8) *Cinnamomum cassia* Presl (9) *Ligustrum lucidum* Ait. (10) *Cinnamomum cassia* Presl	(1) Aconiti lateralis radix preparata (2) Fresh Mongolian milkvetch root (3) Salvia miltiorrhiza (4) White peony root (5) Pubescentholly root (6) Plantain seed (7) Semen descurainiae lepidii (8) Cinnamon (9) Glossy privet fruit (10) Cassia twig	(1) Fuzi (2) Huangqi (3) Dangshen (4) Baishao (5) Maodongqing (6) Cheqianzi (7) Tinglizi (8) Rougui (9) Nvzhenzi (10) Guizhi	11 g per pack

Zou [18]	Nuanxin capsule	(1) *Panax ginseng* C.A. Mey. (2) *Aconitum carmichaelii* Debx. (3) *Coix lacryma*-jobi L.var.ma-yuen (Roman.) Stapf (4) Poria cocos (Schw.) Wolf (5) Pinellia ternate (Thunb.) Breit. (6) *Citrus reticulata* Blanco and *Citrus reticulate* “dahongpao” or *Citrus reticulata* Blanco “Tangerina” (7) *Panax notoginseng* (Burk.) F. H. Chen	(1) Radix ginseng rubra (2) Aconiti lateralis radix preparata (3) Coix seed (4) Tuckahoe (5) Rhizoma Pinelliae Preparata (6) Tangerine peel (7) Sanchi	(1) Hongshen (2) Shufuzi (3) Yiyiren (4) Fuling (5) Fabanxia (6) Juhong (7) Sanqi	0.45 g per capsule

Liu et al. [20]	Nuanxin capsule	(1) *Panax ginseng* C.A. Mey. (2) *Aconitum carmichaelii* Debx. (3) *Coix lacryma*-jobi L.var.ma-yuen (Roman.) Stapf (4) *Poria cocos* (Schw.) Wolf (5) *Pinellia ternate* (Thunb.) Breit. (6) *Citrus reticulata* Blanco and *Citrus reticulate* “Dahongpao” Or *Citrus reticulata* Blanco “Tangerina” (7) *Panax notoginseng* (Burk.) F.H. Chen	(1) Radix ginseng rubra (2) Aconiti lateralis radix preparata (3) Coix seed (4) Tuckahoe (5) Rhizoma Pinelliae Preparata (6) Tangerine peel (7) Sanchi	(1) Hongshen (2) Shufuzi (3) Yiyiren (4) Fuling (5) Fabanxia (6) Juhong (7) Sanqi	0.45 g per capsule

Wang [21]	Tongyang Huoxue granule	(1) *Aconitum carmichaelii* Debx. (2) *Zingiber officinale* Rosc. (3) *Astragalus membranaceus* (Fisch.) Bge.var.mongholicus (Bge.) Hsiao and *Astragalus membranaceus* (Fisch.) Bge. (4) *Salvia miltiorrhiza* Bge.	(1) Aconiti lateralis radix preparata (2) Zingiberis Rhizoma (3) Fresh Mongolian milkvetch root (4) Salvia miltiorrhiza	(1) Paofuzi (2) Ganjiang (3) Huangqi (4) Danshen	9 g per granule

Cao et al. [22]	Kangshuai oral solution	(1) *Panax ginseng* C.A. Mey. (2) *Aconitum carmichaelii* Debx. (3) *Salvia miltiorrhiza* Bge. (4) *Acanthopanax gracilistylus* W. W. Smith	(1) Ginseng radix et rhizoma (2) Aconiti lateralis radix preparata (3) Salvia miltiorrhiza (4) Acanthopanacis Cortex	(1) Renshen (2) Fupian (3) Danshen (4) Wujiapi	Not mentioned

Zou et al. [23]	Nuanxin capsule	(1) *Panax ginseng* C.A. Mey. (2) *Aconitum carmichaelii* Debx. (3) *Coix lacryma*-jobi L.var.ma-yuen (Roman.)Stapf (4) *Poria cocos* (Schw.) Wolf (5) *Pinellia ternate* (Thunb.) Breit. (6) *Citrus reticulata* Blanco and *Citrus reticulate* “Dahongpao” Or *Citrus reticulata* Blanco “Tangerina” (7) *Panax notoginseng* (Burk.) F.H. Chen	(1) Radix ginseng rubra (2) Aconiti lateralis radix preparata (3) Coix seed (4) Tuckahoe (5) Rhizoma Pinelliae Preparata (6) Tangerine peel (7) Sanchi	(1) Hongshen (2) Shufuzi (3) Yiyiren (4) Fuling (5) Fabanxia (6) Juhong (7) Sanqi	0.45 g per capsule
Li et al. [24]	Qili qiangxin capsule	(1) *Astragalus membranaceus* (Fisch.) Bge.var.mongholicus (Bge.) Hsiao and *Astragalus membranaceus* (Fisch.) Bge. (2) *Panax ginseng* C.A. Mey. (3) *Aconitum carmichaelii* Debx. (4) *Salvia miltiorrhiza* Bge. (5) *Descurainia sophia* (L.) Webb.ex Prantl. (6) *Rhizoma alismatis* (7) *Alisma orientalis* (Sam.) Juzep. (8) *Polygonatum odoratum* (Mill.) Druce (9) *Carthamus tinctorius* L. (10) *Periploca sepium* Bge. (11) *Citrus reticulata* Blanco and *Citrus reticulate* “Chachi” Or *Citrus reticulate* “Dahongpao” or *Citrus reticulata* “Unshiu” Or Citrus reticulata “Tangerina”	(1) Fresh Mongolian milkvetch root (2) Ginseng radix et rhizoma (3) Aconiti lateralis radix preparata (4) Salvia miltiorrhiza (5) Semen descurainiae lepidii (6) Alismatis rhizoma (7) Polygonati odotati rhizoma (8) Cinnamomi ramulus (9) Carthami flos (10) Periploca cortex (11) Citri reticulatae pericarpium	(1) Haungqi (2) Renshen (3) Heishunpian (4) Danshen (5) Tinglizi (6) Zexie (7) Yuzhu (8) Guizhi (9) Honghua (10) Xiangjiapi (11) Chenpi	0.3 g per capsule

Dong [25]	Shenfu qiangxin pill	(1) *Panax ginseng* C.A. Mey. (2) *Aconitum carmichaelii* Debx. (3) *Descurainia sophia* (L.) Webb.ex Prantl. (4) *Morus alba* L. (5) Rheum palmatum L.and Rheum tanguticum Maxim.ex Balf. and Rheum officinale Braill. (6) Polyporus umbellatus (Pers.) Fries	(1) Ginseng radix et rhizoma (2) Aconiti lateralis radix preparata (3) Semen descurainiae lepidii (4) Mori Cortex (5) Rhei Radix et Rhizoma (6) Polyporus	(1) Renshen (2) Fuzi (3) Tinglizi (4) Sangbaipi (5) Dahuang (6) Zhuling	3 g per pill

Li et al. [26]	Baoyuan shipi decoction	(1) *Panax ginseng* C.A. Mey. (2) *Astragalus membranaceus* (Fisch.) Bge.var.mongholicus (Bge.) Hsiao and *Astragalus membranaceus* (Fisch.) Bge. (3) *Glycyrrhiza uralensis* Fisch. *Glycyrrhiza inflate* Bat. Glycyrrhiza glabra L. (4) *Magnolia officinalis* Rehd.et Wils. or *Magnolia officinalis* Rehd.et Wils.Var.bilobaReld.et Wils. (5) *Atractylodes macrocephala* Koidz (6) *Chaenomeles speciose* (sweet) Nakai (7) Amomum tsao-ko Crevost et Lemaire (8) *Areca catechu* L. (9) *Aconitum carmichaelii* Debx. (10) *Poria cocos* (Schw.) Wolf (11) *Zingiber officinale* Rosc.	(1) Ginseng radix et rhizoma (2) Fresh Mongolian milkvetch root (3) Radix glycyrrhizae prepared (4) Magnoliae Officmalis Cortex (5) Large head Atractylodes Rhizome (6) Chaenomelis Fructus (7) Fructus Tsaoko (8) Betelnut peel (9) Aconiti lateralis radix preparata (10) Tuckahoe (11) Zingiberis Rhizoma	(1) Renshen (2) Huangqi (3) Zhigancao (4) Houpu (5) Baizhu (6) Mugua (7) Caoguoren (8) Dafupi (9) Fuzi (10) Fuling (11) Ganjiang	Not mentioned

Wei [27]	Wenyang lishui decoction	(1) Ginseng radix et rhizoma (2) *Poria cocos* (Schw.) Wolf (3) *Cinnamomum cassia* Presl (4) *Aconitum carmichaelii* Debx. (5) *Atractylodes macrocephala* Koidz (6) Crataegus pinnatifida Bge.var.major N.E.Br.or Crataegus pinnatifida Bge. (7) *Salvia miltiorrhiza* Bge. (8) *Glycyrrhiza uralensis* Fisch. *Glycyrrhiza inflate* Bat. *Glycyrrhiza glabra* L.	(1) Radix Salviae Miltiorrhizae (2) Tuckahoe (3) Cinnamomi ramulus (4) Typhonii Rhizoma (5) Rhizoma artactylodis macrocephalae (6) Hawthorn fruit (7) Salvia miltiorrhiza (8) Radix Rhizoma Glycyrrhizae	(1) Shengshaishen (2) Fuling (3) Guizhi (4) Baifupian (5) Chaobaizhu (6) Shanzha (7) Danshen (8) Shenggancao	15 15 15 15 15 20 20 5g
Zhu [28]	Yiqi qiangxin decoction	(1) *Codonopsis pilosula* (Franch.) Nannf. Or *Codonopsis pilosula* Nannf.var.modesta (Nannf.) L.T. Shen Or *Codonopsis tangshen* Oliv. (2) *Astragalus membranaceus* (Fisch.) Bge.var.mongholicus (Bge.) Hsiao and *Astragalus membranaceus* (Fisch.) Bge. (3) *Angelica sinensis* (Oliv.) Diels (4) *Pheretima aspergillum* (E. Perrier) or Pheretima vulgaris Chen Or *Pheretima guillelmi* (Michaelsen) or *Pheretima pectinifera* Michaelsen (5) *Crataegus pinnatifida* Bge.var.major N.E.Br. or *Crataegus pinnatifida* Bge. (6) *Poria cocos* (Schw.) Wolf (7) *Glycyrrhiza uralensis* Fisch. and *Glycyrrhiza inflate* Bat. and *Glycyrrhiza glabra* L. (8) *Leonurus japonicus* Houtt. (9) *Aconitum carmichaelii* Debx. (10) *Paeonia lactiflora* Pall. (11) *Descurainia sophia* (L.) Webb.ex Prantl. (12) *Salvia miltiorrhiza* Bge.	(1) Root of Pilose Asiabell (2) Fresh Mongolian milkvetch root (3) Angelicae Sinensis Radix (4) Pheretima (5) Rhizoma artactylodis macrocephalae (6) Tuckahoe (7) Radix glycyrrhizae prepared (8) Herba Leonuri (9) Aconiti lateralis radix preparata (10) White peony root (11) Semen descurainiae lepidii (12) Salvia miltiorrhiza	(1) Dangshen (2) Shenghuangqi (3) Danggui (4) Dilong (5) Chaobaizhu (6) Fuling (7) Zhigancao (8) Yimucao (9) Fuzi (10) Baishao (11) Tinglizi (12) Danshen	30 50 20 20 20 20 15 20 15 20 15 20

Wang et al. [29]	SFJ	(1) *Panax ginseng* C.A. Mey. (2) *Aconitum carmichaelii* Debx.	(1) Ginseng radix et rhizome (2) Aconiti lateralis radix preparata	(1) Renshen (2) Fuzi	Not mentioned

SFJ = Shenfu injection.

**Table 3:** The top 6 frequently used Chinese herbs in FZF formulae.

Latin name	English name	Chinese name	Frequency	The total frequency (%)
1. *Aconitum carmichaelii* Debx 2. *Panax ginseng* C.A. Mey 3. *Salvia miltiorrhiza* Bge 4. *Poria cocos* (Schw.) Wolf 5. *Astragalus membranaceus* (Fisch.) Bge.var.mongholicus (Bge.) Hsiao and *Astragalus membranaceus* (Fisch.) Bge 6. *Descurainia sophia* (L.) Webb.ex Prantl.	(1) Aconiti lateralis radix preparata (2) Ginseng radix et rhizome (3) Salvia miltiorrhiza (4) Tuckahoe (5) Fresh Mongolian milkvetch root (6) Semen descurainiae lepidii	(1) Fuzi (2) Renshen (3) Danshen (4) Fuling (5) Huangqi (6) Tinglizi	12 9 7 6 5 4	40 30 23.3 20 16.7 13.3

**Table 4:** Risk of bias summary and scores of included studies.

Studies	A	B	C	D	E	F	G	Scores
Huo et al. [19]	+	+	+	?	+	+	+	6
Zou [18]	+	?	?	-	+	+	+	4
Liu et al. [20]	+	?	+	+	+	?	+	4
Wang [21]	+	?	-	?	+	+	+	4
Cao et al. [22]	+	?	+	?	+	+	+	6
Zou et al. [23]	+	+	+	+	+	+	+	6
Li et al. [24]	+	+	+	?	+	+	+	6
Dong [25]	+	?	-	?	+	+	+	4
Li et al. [26]	+	?	+	?	+	+	+	4
Wei [27]	+	?	-	?	+	+	+	4
Zhu [28]	+	?	-	?	+	+	+	4
Wang et al. [29]	+	+	+	?	+	+	+	5

A, random sequence generation (selection bias); B, allocation concealment (selection bias); C, blinding of participants and personnel (performance bias); D, blinding of outcome assessment (detection bias); E, incomplete outcome data (attrition bias); F, selective reporting (reporting bias); G, other bias.+, low risk of bias;-, high risk of bias; ?, unclear risk of bias.

**Table 5:** Statement of facts (SoF) table for first outcomes.

Primary outcomes of the treatment of heart failure as complementary therapy: a systematic review and meta-analysis of high-quality randomized controlled trials						
Patient or population: patients with the treatment of heart failure as complementary therapy: a systematic review and meta-analysis of high-quality randomized controlled trials Intervention: primary outcomes						

Outcomes	Illustrative comparative risks^*∗*^ (95% CI)		Relative effect (95% CI)	No. of participants (studies)	Quality of the evidence (GRADE)	Comments
	Assumed risk	Corresponding risk				
	Control	Primary outcomes				

*Plasma NT-proBNP level* Enzyme-linked immunosorbent assay. Scale from 1 to 35000. Follow-up: 10–168 days	The mean plasma NT-proBNP level ranged across control groups from 403.5 to 4455.8 pg/ml	The mean plasma NT-proBNP level in the intervention groups was 1.76 standard deviations lower (2.87 to 0.66 lower)		483 (6 studies)	⊕⊕⊝⊝ low	SMD −1.76 (−2.87 to −0.66)

*Efficacy on TCM* Guiding Principles of clinical Research on new Chinese medicine for heart failure Follow-up: 6–168 days	Study population		RR 1.37 (1.26 to 1.48)	760 (7 studies)	⊕⊕⊕⊝ moderate	
	638 per 1000	874 per 1000 (804 to 945)				
	Moderate					
	620 per 1000	849 per 1000 (781 to 918)				

*Efficacy on TCMS-FZF plus CHFST vs CHFST* Follow-up: 10–84 days	Study population		RR 1.35 (1.22 to 1.48)	472 (5 studies)	⊕⊕⊕⊝ moderate	
	652 per 1000	881 per 1000 (796 to 965)				
	Moderate					
	620 per 1000	837 per 1000 (756 to 918)				

*Efficacy of TCMS-FZF plus CHFST vs placebo plus CHFST* Follow-up: 6–168 days	Study population		RR 1.42 (1.23 to 1.64)	288 (2 studies)	⊕⊕⊕⊕ high	
	615 per 1000	874 per 1000 (757 to 1000)				
	Moderate					
	615 per 1000	873 per 1000 (756 to 1000)				

^*∗*^The basis for the assumed risk (e.g., the median control group risk across studies) is provided. The corresponding risk (and its 95% confidence interval) is based on the assumed risk in the comparison group and the relative effect of the intervention (and its 95% CI). CI: confidence interval; RR: risk ratio. GRADE working group grades of evidence: high quality: further research is very unlikely to change our confidence in the estimate of effect; moderate quality: further research is likely to have an important impact on our confidence in the estimate of effect and may change the estimate; low quality: further research is very likely to have an important impact on our confidence in the estimate of effect and is likely to change the estimate; very low quality: we are very uncertain about the estimate.

**Table 6:** Statement of facts (SoF) table for secondary outcomes.

Secondary outcomes of the treatment of heart failure as complementary therapy: a systematic review and meta-analysis of high-quality randomized controlled trials						
Patient or population: patients with the treatment of heart failure as complementary therapy: a systematic review and meta-analysis of high-quality randomized controlled trials Settings: Intervention: secondary outcomes						

Outcomes	Illustrative comparative risks^*∗*^ (95% CI)		Relative effect (95% CI)	No. of participants (studies)	Quality of the evidence (GRADE)	Comments
	Assumed risk	Corresponding risk				
	Control	Secondary outcomes				

*NYHA functional classification (NYHAfc)* 1982 American New York Heart Association (NYHA) Follow-up: 6–252 days	Study population		RR 1.31 (1.21 to 1.41)	848 (9 studies)	⊕⊕⊕⊝ moderate	
	653 per 1000	856 per 1000 (790 to 921)				
	Moderate					
	733 per 1000	960 per 1000 (887 to 1000)				

*NYHA functional classification (NYHAfc)-FZF plus CHFST vs CHFST* 1982 American New York Heart Association (NYHA) Follow-up: 10–168 days	Study population		RR 1.37 (1.24 to 1.52)	483 (6 studies)	⊕⊕⊝⊝ low	
	646 per 1000	885 per 1000 (801 to 982)				
	Moderate					
	733 per 1000	1000 per 1000 (909 to 1000)				

*NYHA functional classification (NYHAfc)-FZF plus CHFST vs placebo plus CHFST* 1982 American New York Heart Association (NYHA) Follow-up: 6–252 days	Study population		RR 1.22 (1.08 to 1.38)	365 (3 studies)	⊕⊕⊕⊝ moderate	
	663 per 1000	809 per 1000 (716 to 915)				
	Moderate					
	644 per 1000	786 per 1000 (696 to 889)				

*LVEF* Simpson. Scale from 0 to 100 Follow-up: 6–168 days	The mean LVEF ranged across control groups from 28.25 to 46.17 percentage	The mean LVEF in the intervention groups was 0.48 standard deviations higher (0.03 to 0.94 higher)		1088 (7 studies)	⊕⊕⊝⊝ low	SMD 0.48 (0.03 to 0.94)

*LVEF-FZF plus CHFST vs CHFST* Simpson. Scale from 0 to 100. Follow-up: 14–168 days	The mean LVEF-FZF plus CHFST vs CHFST ranged across control groups from 41.7 to 46.17 percentage	The mean LVEF-FZF plus CHFST vs CHFST in the intervention groups was 0.98 standard deviations higher (0.42 to 1.54 higher)		322 (4 studies)	⊕⊝⊝⊝ very low	SMD 0.98 (0.42 to 1.54)

*LVEF-FZF plus CHFST vs placebo plus CHFST* Simpson. Scale from 0 to 100. Follow-up: 6–168 days	The mean LVEF-FZF plus CHFST vs placebo plus CHFST ranged across control groups from 28.25 to 39.82 percentage	The mean LVEF-FZF plus CHFST vs placebo plus CHFST in the intervention groups was 0.1 standard deviations lower (0.5 lower to 0.3 higher)		766 (3 studies)	⊕⊕⊕⊝ moderate	SMD −0.1 (−0.5 to 0.3)

*6MWD* 6MWT. Scale from 0 to 1000. Follow-up: 6–252 days	The mean 6MWD ranged across control groups from 82.99 to 405.97 meter	The mean 6MWD in the intervention groups was 0.55 standard deviations higher (0.39 to 0.72 higher)		1168 (7 studies)	⊕⊕⊕⊝ moderate	SMD 0.55 (0.39 to 0.72)

*6MWD-FZF plus CHFST vs CHFST 6MWT*. Scale from 0 to 1000 Follow-up: 6–168 days	The mean 6MWD-FZF plus CHFST vs CHFST ranged across control groups from 82.99 to 405.97 meter	The mean 6MWD-FZF plus CHFST vs CHFST in the intervention groups was 0.6 standard deviations higher (0.34 to 0.85 higher)		461 (5 studies)	⊕⊕⊕⊝ moderate	SMD 0.6 (0.34 to 0.85)

*6MWD-FZF plus CHFST vs placebo plus CHFST* 6MWT. Scale from 0 to 1000. Follow-up: 6–252 days	The mean 6MWD-FZF plus CHFST vs placebo plus CHFST ranged across control groups from 82.99 to 368.08 meter	The mean 6MWD-FZF plus CHFST vs placebo plus CHFST in the intervention groups was 0.52 standard deviations higher (0.25 to 0.78 higher)		707 (3 studies)	⊕⊕⊕⊝ moderate	SMD 0.52 (0.25 to 0.78)

*MLHFQ scores and Lee's heart failure scores* Minnesota heart failure quality of life scale. Scale from 0 to 50. Follow-up: 10–84 days	The mean MLHFQ scores and Lee's heart failure scores ranged across control groups from 1.33 to 43.13 points	The mean MLHFQ scores and Lee's heart failure scores in the intervention groups was 0.57 standard deviations lower (0.75 to 0.39 lower)		495 (4 studies)	⊕⊕⊕⊝ moderate	SMD −0.57 (−0.75 to −0.39)

*MLHFQ scores and Lee's heart failure scores-MLHFQ scores* Minnesota heart failure quality of life scale Follow-up: 14–84 days		The mean MLHFQ scores and Lee's heart failure scores-MLHFQ scores in the intervention groups was 0.61 standard deviations lower (0.88 to 0.34 lower)		227 (3 studies)	⊕⊕⊝⊝ low^1^	SMD −0.61 (−0.88 to −0.34)

MLHFQ *scores and Lee's heart failure scores-Lee's heart failure scores* Minnesota heart failure quality of life scale. Scale from 0 to 50. Follow-up: 10–82 days	The mean MLHFQ scores and Lee's heart failure scores-Lee's heart failure scores ranged across control groups from 1.33 to 6.58 points	The mean MLHFQ scores and Lee's heart failure scores-Lee's heart failure scores in the intervention groups was 0.53 standard deviations lower (0.78 to 0.29 lower)		268 (3 studies)	⊕⊕⊝⊝ low	SMD −0.53 (−0.78 to −0.29)

*CCEs* Death and readmission Follow-up: 6–252 days	Study population		RR 0.45 (0.33 to 0.61)	1568 (4 studies)	⊕⊕⊕⊕ high	
	130 per 1000	58 per 1000 (43 to 79)				
	Moderate					
	184 per 1000	83 per 1000 (61 to 112)				

*CCEs-deaths* Death Follow-up: 6–252 days	Study population		RR 0.33 (0.17 to 0.64)	856 (4 studies)	⊕⊕⊕⊕ high	
	75 per 1000	25 per 1000 (13 to 48)				
	Moderate					
	106 per 1000	35 per 1000 (18 to 68)				

*CCEs-readmission for heart failure* Readmission for heart failure Follow-up: 84–252 days	Study population		RR 0.48 (0.34 to 0.67)	712 (3 studies)	⊕⊕⊕⊕ high	
	201 per 1000	97 per 1000 (68 to 135)				
	Moderate					
	447 per 1000	215 per 1000 (152 to 299)				

^*∗*^The basis for the assumed risk (e.g., the median control group risk across studies) is provided. The corresponding risk (and its 95% confidence interval) is based on the assumed risk in the comparison group and the relative effect of the intervention (and its 95% CI). CI: confidence interval; RR: risk ratio. GRADE working group grades of evidence: high quality: further research is very unlikely to change our confidence in the estimate of effect; moderate quality: further research is likely to have an important impact on our confidence in the estimate of effect and may change the estimate; low quality: further research is very likely to have an important impact on our confidence in the estimate of effect and is likely to change the estimate; very low quality: we are very uncertain about the estimate.

**Table 7:** Summary of adverse events.

Studies	Experimental		Control		AEs
	*n* (case)	Total	*n* (case)	Total	
Huo et al. [19]		48	1	47	Erythra
Zou [18]		59	1	60	Chest tightness and heart palpitations
Liu et al. [20]		30	1	30	Erythra
Zou et al. [23]	1	71		73	Cough
Li et al. [24]	66	244	98	247	Not mentioned in detail
Wang et al. [29]	2	78	0	79	Erythra, chills

AE: adverse event.

## Supplementary Materials

Supplementary Materials Appendix 1: the search strategy of PubMed was as follows: #1 Search ((((((((((((((Heart failure[Title/Abstract]) OR Cardiac Failure[Title/Abstract]) OR Heart Decompensation[Title/Abstract]) OR Decompensation, Heart[Title/Abstract]) OR Heart Failure, Right-Sided[Title/Abstract]) OR Heart Failure, Right Sided[Title/Abstract]) OR Right-Sided Heart Failure[Title/Abstract]) OR Right Sided Heart Failure[Title/Abstract]) OR Myocardial Failure[Title/Abstract]) OR Congestive Heart Failure[Title/Abstract]) OR Heart Failure, Congestive[Title/Abstract]) OR Heart Failure, Left-Sided[Title/Abstract]) OR Heart Failure, Left Sided[Title/Abstract]) OR Left-Sided Heart Failure[Title/Abstract]) OR Left Sided Heart Failure[Title/Abstract] #2 Search (((((((((((((Traditional Chinese medicine[Title/Abstract]) OR Chung I Hsueh[Title/Abstract]) OR Hsueh, Chung I[Title/Abstract]) OR Traditional Medicine, Chinese[Title/Abstract]) OR Zhong Yi Xue[Title/Abstract]) OR Chinese Traditional Medicine[Title/Abstract]) OR Chinese Medicine, Traditional[Title/Abstract]) OR Chinese Medicine, Traditional[Title/Abstract]) OR Tongue Diagnoses, Traditional[Title/Abstract]) OR Tongue Diagnosis, Traditional[Title/Abstract]) OR Traditional Tongue Diagnoses[Title/Abstract]) OR Traditional Tongue Assessment[Title/Abstract]) OR Tongue Assessment, Traditional[Title/Abstract]) OR Traditional Tongue Assessments[Title/Abstract] #1 AND #2. Appendix 2: Step 1: design excel for each outcome. Step 2: import data form included trails into excel.
