# Supplementary material for: Efficacy and Safety of Fuzi Formulae on the Treatment of Heart Failure as Complementary Therapy: A Systematic Review and Meta-Analysis of High-Quality Randomized Controlled Trials
Source: Evid Based Complement Alternat Med. 2019 Dec 23;2019:9728957. doi: 10.1155/2019/9728957 (PMC6948335; doi:10.1155/2019/9728957)

Appendix1

The search strategy of PubMed was as follows:

#1 Search (((((((((((((Heart failure[Title/Abstract]) OR Cardiac Failure[Title/Abstract]) OR Heart Decompensation[Title/Abstract]) OR Decompensation, Heart[Title/Abstract]) OR Heart Failure, Right-Sided[Title/Abstract]) OR Heart Failure, Right Sided[Title/Abstract]) OR Right-Sided Heart Failure[Title/Abstract]) OR Right Sided Heart Failure[Title/Abstract]) OR Myocardial Failure[Title/Abstract]) OR Congestive Heart Failure[Title/Abstract]) OR Heart Failure, Congestive[Title/Abstract]) OR Heart Failure, Left-Sided[Title/Abstract]) OR Heart Failure, Left Sided[Title/Abstract]) OR Left-Sided Heart Failure[Title/Abstract]) OR Left Sided Heart Failure[Title/Abstract]

#2 Search ((((((((((((Traditional Chinese medicine[Title/Abstract]) OR Chung I Hsueh[Title/Abstract]) OR Hsueh, Chung I[Title/Abstract]) OR Traditional Medicine, Chinese[Title/Abstract]) OR Zhong Yi Xue[Title/Abstract]) OR Chinese Traditional Medicine[Title/Abstract]) OR Chinese Medicine, Traditional[Title/Abstract]) OR Chinese Medicine, Traditional[Title/Abstract]) OR Tongue Diagnoses, Traditional[Title/Abstract]) OR Tongue Diagnosis, Traditional[Title/Abstract]) OR Traditional Tongue Diagnoses[Title/Abstract]) OR Traditional Tongue Assessment[Title/Abstract]) OR Tongue Assessment, Traditional[Title/Abstract]) OR Traditional Tongue Assessments[Title/Abstract]

#1 AND #2

Appendix2

Step1: Design excel for each outcome

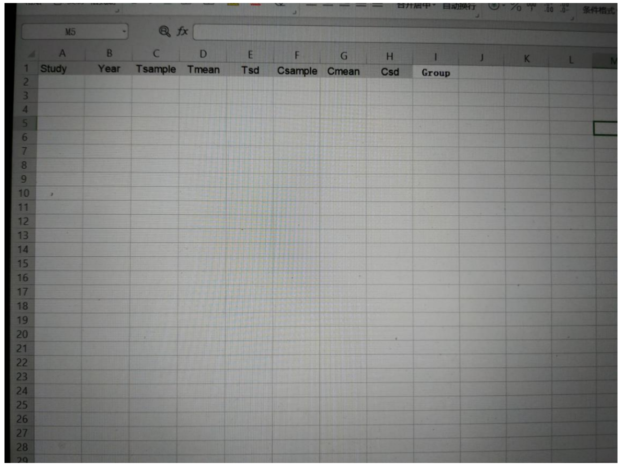

Step2:Import data form included trails into excel

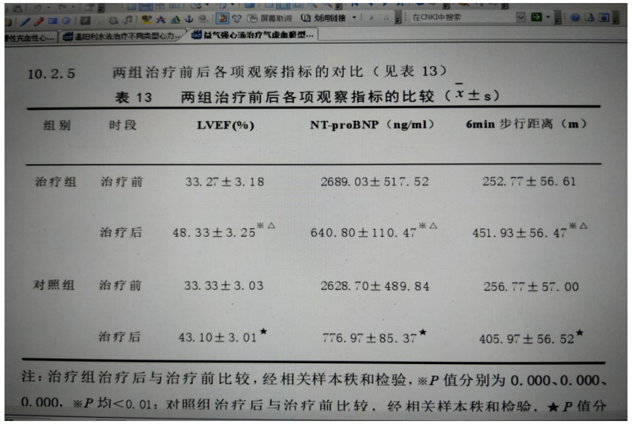

10.2.5　两组治疗前后各项观察指标的对比（见表 13）

表 13　两组治疗前后各项观察指标的比较（$\bar{x} \pm s$）

组别	时段	LVEF(%)	NT-proBNP（ng/ml）	6min 步行距离（m）
治疗组	治疗前	33.27 ± 3.18	2689.03 ± 517.52	252.77 ± 56.61
	治疗后	$48.33 \pm 3.25^{※\triangle}$	$640.80 \pm 110.47^{※\triangle}$	$451.93 \pm 56.47^{※\triangle}$
对照组	治疗前	33.33 ± 3.03	2628.70 ± 489.84	256.77 ± 57.00
	治疗后	$43.10 \pm 3.01^{\star}$	$776.97 \pm 85.37^{\star}$	$405.97 \pm 56.52^{\star}$

注：治疗组治疗后与治疗前比较，经相关样本秩和检验，※P 值分别为 0.000、0.000、0.000．※P 均<0.01；对照组治疗后与治疗前比较，经相关样本秩和检验，★P 值分

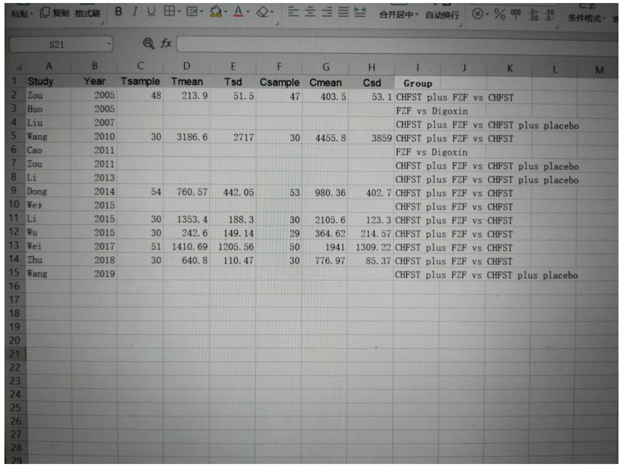

	A	B	C	D	E	F	G	H	I
1	Study	Year	Tsample	Tmean	Tsd	Csample	Cmean	Csd	Group
2	Zou	2005	48	213.9	51.5	47	403.5	53.1	CHFST plus FZF vs CHFST
3	Huo	2005							FZF vs Digoxin
4	Liu	2007							CHFST plus FZF vs CHFST plus placebo
5	Wang	2010	30	3186.6	2717	30	4455.8	3859	CHFST plus FZF vs CHFST
6	Cao	2011							FZF vs Digoxin
7	Zou	2011							CHFST plus FZF vs CHFST plus placebo
8	Li	2013							CHFST plus FZF vs CHFST plus placebo
9	Dong	2014	54	760.57	442.05	53	980.36	402.7	CHFST plus FZF vs CHFST
10	Wei	2015							CHFST plus FZF vs CHFST
11	Li	2015	30	1353.4	188.3	30	2105.6	123.3	CHFST plus FZF vs CHFST
12	Wu	2015	30	242.6	149.14	29	364.62	214.57	CHFST plus FZF vs CHFST
13	Wei	2017	51	1410.69	1205.56	50	1941	1309.22	CHFST plus FZF vs CHFST
14	Zhu	2018	30	640.8	110.47	30	776.97	85.37	CHFST plus FZF vs CHFST
15	Wang	2019							CHFST plus FZF vs CHFST plus placebo

Step3:Import the excel into Review Manager2 software, use random effect model to calculate the effective rate

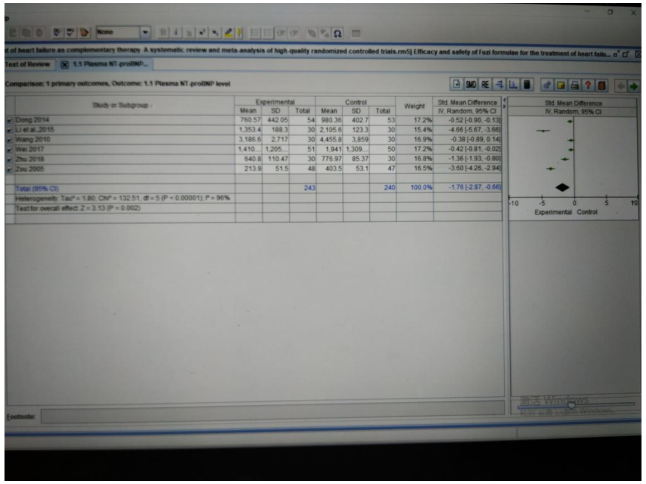

Comparison: 1 primary outcomes, Outcome: 1.1 Plasma NT-proBNP level

Study or Subgroup	Experimental Mean	Experimental SD	Experimental Total	Control Mean	Control SD	Control Total	Weight	Std. Mean Difference IV, Random, 95% CI
Dong 2014	760.57	442.05	54	980.36	402.7	53	17.2%	-0.52 [-0.90, -0.13]
Li et al.,2015	1,353.4	188.3	30	2,105.6	123.3	30	15.4%	-4.66 [-5.67, -3.66]
Wang 2010	3,186.6	2,717	30	4,455.8	3,859	30	16.9%	-0.38 [-0.89, 0.14]
Wei 2017	1,410…	1,205…	51	1,941	1,309…	50	17.2%	-0.42 [-0.81, -0.02]
Zhu 2018	640.8	110.47	30	776.97	85.37	30	16.8%	-1.36 [-1.93, -0.80]
Zou 2005	213.9	51.5	48	403.5	53.1	47	16.5%	-3.60 [-4.26, -2.94]
Total (95% CI)			243			240	100.0%	-1.76 [-2.87, -0.66]
Heterogeneity: $Tau^2 = 1.80$; $Chi^2 = 132.51$, $df = 5$ ($P < 0.00001$); $I^2 = 96\%$								
Test for overall effect: $Z = 3.13$ ($P = 0.002$)								

Step4: Finally, we could see the risk ratio from the forest plot

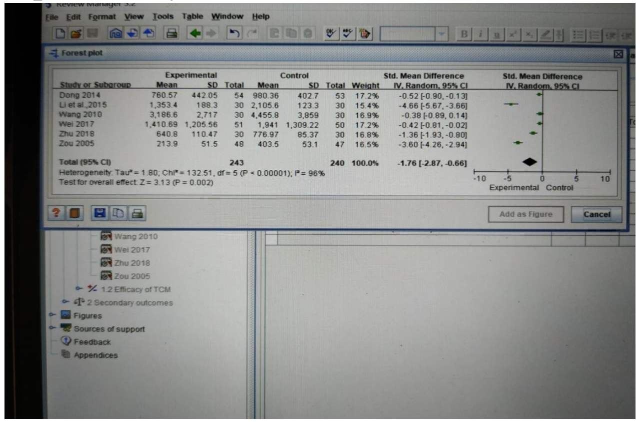

Supplement: Supplementary Materials — Appendix 1: the search strategy of PubMed was as follows: #1 Search ((((((((((((((Heart failure[Title/Abstract]) OR Cardiac Failure[Title/Abstract]) OR Heart Decompensation[Title/Abstract]) OR Decompensation, Heart[Title/Abstract]) OR Heart Failure, Right-Sided[Title/Abstract]) OR Heart Failure, Right Sided[Title/Abstract]) OR Right-Sided Heart Failure[Title/Abstract]) OR Right Sided Heart Failure[Title/Abstract]) OR Myocardial Failure[Title/Abstract]) OR Congestive Heart Failure[Title/Abstract]) OR Heart Failure, Congestive[Title/Abstract]) OR Heart Failure, Left-Sided[Title/Abstract]) OR Heart Failure, Left Sided[Title/Abstract]) OR Left-Sided Heart Failure[Title/Abstract]) OR Left Sided Heart Failure[Title/Abstract] #2 Search (((((((((((((Traditional Chinese medicine[Title/Abstract]) OR Chung I Hsueh[Title/Abstract]) OR Hsueh, Chung I[Title/Abstract]) OR Traditional Medicine, Chinese[Title/Abstract]) OR Zhong Yi Xue[Title/Abstract]) OR Chinese Traditional Medicine[Title/Abstract]) OR Chinese Medicine, Traditional[Title/Abstract]) OR Chinese Medicine, Traditional[Title/Abstract]) OR Tongue Diagnoses, Traditional[Title/Abstract]) OR Tongue Diagnosis, Traditional[Title/Abstract]) OR Traditional Tongue Diagnoses[Title/Abstract]) OR Traditional Tongue Assessment[Title/Abstract]) OR Tongue Assessment, Traditional[Title/Abstract]) OR Traditional Tongue Assessments[Title/Abstract] #1 AND #2. Appendix 2: Step 1: design excel for each outcome. Step 2: import data form included trails into excel. [file 9728957.f1.pdf]
